# An Evolutionary Game-Theoretic Approach for Assessing Privacy Protection in mHealth Systems

**DOI:** 10.3390/ijerph15102196

**Published:** 2018-10-08

**Authors:** Guang Zhu, Hu Liu, Mining Feng

**Affiliations:** 1 School of Management Science and Engineering, Nanjing University of Information Science and Technology, Nanjing 210044, China; 18752099127@163.com; 2 China Institute of Manufacturing Development, Nanjing University of Information Science and Technology, Nanjing 210044, China; fengmn_nuist@163.com

**Keywords:** mHealth, privacy protection, investment, evolutionary game, free riding, regulation

## Abstract

With the rapid deployment of mobile technologies and their applications in the healthcare domain, privacy concerns have emerged as one of the most critical issues. Traditional technical and organizational approaches used to address privacy issues ignore economic factors, which are increasingly important in the investment strategy of those responsible for ensuring privacy protection. Taking the mHealth system as the context, this article builds an evolutionary game to model three types of entities (including system providers, hospitals and governments) under the conditions of incomplete information and bounded rationality. Given that the various participating entities are often unable to accurately estimate their own profits or costs, we propose a quantified approach to analyzing the optimal strategy of privacy investment and regulation. Numerical examples are provided for illustration and simulation purpose. Based upon these examples, several countermeasures and suggestions for privacy protection are proposed. Our analytical results show that governmental regulation and auditing has a significant impact on the strategic choice of the other two entities involved. In addition, the strategic choices of system providers and hospitals are not only correlated with profits and investment costs, but they are also significantly affected by free riding. If the profit growth coefficients increase to a critical level, mHealth system providers and hospitals will invest in privacy protection even without the imposition of regulations. However, the critical level is dependent on the values of the parameters (variables) in each case of investment and profits.

## 1. Introduction

Recently, the significant advances in Internet and mobile communications have had a great impact on wireless networks and mobile applications [1]. By enabling patients to manage their health data (e.g., via electronic health records) more conveniently [2], better tracking of medicine supplies [3] and reducing the cost of care [4], mHealth technology has enormous potential for improving the quality and timely delivery of healthcare [5]. However, mHealth is a double-edged sword technology and its potential benefits have come accompanied by the threat of privacy violations [6]. According to a survey by Healthcare Information and Management System Society (HIMMS), only 38% of clinicians use patients’ Electronic Health Records (EHRs) under a formal privacy policy [7]. The annual Crime Scene Investigation (CSI)/ Federal Bureau of Investigation (FBI) surveys and Computer Emergency Response Team (CERT) statistics show that security breaches have been one of the most significant challenges to OSN. For example, iCloud was attacked by black-hat hackers in 2014; the attack incurred a large data loss that included user identities, emails, and telephone numbers of several million families and firms. SafeNet Corporation reported that during the first half of 2016, 92% of companies and organizations experienced data breaches and that 3,046,456 data records were lost or stolen every day [8]. Therefore, how to develop an effective mechanism for assessing privacy protection in mHealth systems has become a challenge to governments, industries and academia research.

Currently, there are various data protection regulations and cyber security laws in both developed and developing countries. For example, the General Data Protection Regulation (GDPR) is a regulation in EU law on data protection and privacy for all individuals within the European Union (EU) and the European Economic Area (EEA). The GDPR aims primarily to give control to individuals over their personal data and to simplify the regulatory environment for international business by unifying the regulation within the EU [9]. In China, the National Standards on Information Security Technology—Personal Information Security Specification (GB/T 35273-2017) has taken effect on 1 May 2018. The Standard requires transparency, specificity and fairness of processing purpose, proportionality, security, risk assessment, and the respect of individuals’ rights to control the processing of information about them [10]. However, there is still a lack of penalties on privacy violation and data disclosure in China.

In the academia area, the bulk of research on privacy issues in mHealth systems is mainly focused on organizational and technological solutions [11]. However, these types of solutions are no longer sufficient. On the one hand, as communication networks and social media play an increasingly important role, the emergence of new privacy problems might occur. These problems come not only from technological and organizational deficiencies, but also from economical disregard and lack of oversight. On the other hand, profit maximization is considered to be the most important goal of commercial entities. In reality, effective privacy protection in mHealth system requires moderate investment from different entities (e.g., system providers, hospitals, governments and patients). Requirements, may include technological research, equipment purchase, system installation, organizational restructuring, staff training, service fees and/or governmental regulation and auditing [12]. How to balance between the profits from and the costs of privacy investment should be considered. Therefore, privacy-aware entities are shifting their focus from what is technically possible or organizationally do-able, to including what is economically optimal.

The weakness of traditional solutions to privacy concerns is their lack of a quantitative decision framework. Game theory is a branch of applied mathematics that formalizes strategic interaction among autonomous agents, which can provide a mathematical framework for modeling privacy problems in which multiplayers with contradictory or collaborative goals are considered [13]. Furthermore, game theory is capable of analyzing many scenarios before determining the appropriate actions. This can greatly benefit for the entities’ decision making. In recent years, research on game theoretic approaches to security and privacy problems can be organized into six main categories: information security investment, trust and privacy, network security, malicious program, penetration testing and digital forensics [14]. This paper focuses more on information security and privacy investment and network security.

Existing research on game theoretic approaches primarily considers the interaction of two players or two types of players under a competitive scenario [15]. However, three or more types of decision makers might be involved in a game. The relationships between players can be cooperative, selfish, or free riding. Moreover, it is difficult for different entities to identify and achieve the optimal investment strategy in a single game process. Instead, they are assumed to have bounded rationality and to be working under incomplete information. The long-term profit of each stage is different and higher payoff strategies tend to displace lower profit strategies over time. Information security and privacy investment studies based on the theories of a Bayesian game [16], Stackelberg game [17] and differential game [15] cannot solve the above problems, because these games all assume that the game players are rational, and ignore the dynamic process of adapting behavior. Additionally, privacy investment has significant importance in reality and is indispensable for all entities. The co-investment secured by governmental regulation should also be analyzed, but previous scholars have not researched these topics sufficiently.

Evolutionary game theory forgoes a typical assumption of classical game theory: rationality. Instead, evolutionary game theory, supposes that game players (entities) are naïve optimizers operating under imperfect information. Players can adapt their behavior on their immediate context [18]. Entities interact with each other and receive profits based on their strategic choices. Strategies which receive the higher profits spread in the population at the cost of other strategies with lower profits [19]. Evolutionary stability is a refinement of the concept of Nash equilibrium, which leads to ideas such as an “unbeatable strategy” or an “evolutionarily stable strategy (ESS)”. Understanding this dynamic process is the mainstay of evolutionary game theory [20].

This paper applies an evolutionary game theoretic approach to modeling and analyzing the optimal investment strategy of privacy protection. We take the mHealth system as the context, which involves three types of entities: system providers, hospitals and governments. To begin with, we review some assumptions and define several parameters of the evolutionary game model. Then, we analyze the stability of the model, and define reasonable codes of conduct for each player. Furthermore, we provide numerical examples for illustration and to verify the mathematical model. Finally, we suggest several countermeasures that could be used to improve the development of privacy protection in the mHealth system. The main contributions of this paper are as follows:

- We review and summarize the features and weaknesses of existing game theoretical approaches to study privacy related issues.
- We build an evolutionary game model to formulate cooperate interactions and bounded rational confrontation among system providers, hospitals and governments in mHealth system.
- We analyze the different solutions obtained from evolutionary equilibrium and interpret different outcomes on how they may benefit decision makers.
- We construct simulation experiments to prove the usefulness of our proposed model.

The rest of this paper is organized as follows: in Section 2, we review studies that are of relevance. Section 3 describes the decision-making problems of privacy investment, and proposes the evolutionary game model. Then, the ESSs are illustrated under different parameter conditions. Section 4 verifies and analyzes the theoretical results obtained from numerical examples. Section 5 discusses the relationship between the simulation results and strategic choice of privacy investment. Section 6 briefly summarizes our research and provides several future directions.

## 2. Literature Review

Privacy has become one of the most important research issues in the information age [21]. Privacy can be defined as the claim of individuals, groups or organizations to control when, how, and to what extent their personal information is captured or extracted and used by others [22]. Existing scholarly on privacy related issues were examined from three categories–organizational, technical, and economical approaches.

Organizational research is often characterized or related to organizational culture [23], privacy concerns [24], privacy paradox [25] and privacy policies [26]. Technological research mainly focuses on two aspects: anonymity and access control [27]. Anonymity means the hiding and fuzzification of the data source to prevent, associating information with identities of the individuals. The methods of anonymity include handicapping [28], generalization [29], slicing [30], etc. Privacy protection based on access control refers to controlling the network users to access the sensed data, on the one hand, to ensure the legitimate, authenticated, even paid users access the sensitive data efficiently, on the other hand, to prevent illegal, unauthenticated, unpaid users from accessing the nodes resources [31].

Despite being quite comprehensive, the organizational and technological methods of privacy protection have disadvantages and limitations. Effective privacy protection in mHealth systems requires a substantial investment by system providers, hospitals, governments and patients, all of whom also want to maximize their benefits. However, traditional methods ignore the economic factors, such as credit loss, investment costs and expected profits, all of which are important for independent entities attempting to make the optimal strategic decision. Investment decisions in this context lead researchers to use game theoretic approaches to allocate limited resources, model benefit interactions, and take into account the underlying incentive mechanisms [32].

Game theoretic approaches have been proposed by many researchers to improve network security and privacy. The application of game theory in network security and privacy can be classified into several categories: applications for analysis of network attack-defense [33], applications for network security measurement [34], IDS configuration [35], location privacy [36], economics of security and privacy [37], etc. From the perspective of economics, some literature concerning security and privacy investment in information systems has been produced. In [38], a game theoretic approach is applied to address security investment issues, in which the level of profits depends on the interaction between players’ strategic choices. This paper points out that the profits a firm makes from security investment depend on the extent of hacking. In contrast, the hacker’s profits depend on the probability of him or her being caught. In [16], another game-theoretic approach is proposed to investigate different aspects of security investment. Additionally, the potential advantages of using game-theoretic approaches to security investment as opposed to decision-theoretic approaches are discussed. Based on the concepts of Return on Attack (ROA) and Return on Investment (ROI), an attack-defense game tree is used to analyze attack behaviors and the defender’s corresponding strategies [39].

With increasing interdependence, each firm free rides by investing less, and suffers lower profit, while the attacker enjoys higher profit. Therefore, information sharing and cooperation among firms can increase the level of information security; this is consistent to the finding of [40]. In [41], the intrusion detection system (IDS) of OSN is defined as a non-cooperative game, which is used to answer two questions: What are the expected behaviors of rational attackers? What is the optimal strategy for the defenders? The expected behaviors of attackers, the minimum defending resources, and the optimal responding of the defenders are discussed based on a Nash equilibrium analysis. In [42], a game theoretic framework is proposed to model the interaction between small and medium-sized enterprises (SMEs) and attackers and to investigate the allocation of security investment budgets. By emphasizing the importance of security information sharing, a game theoretic model consisting two competitive firms is developed. This research investigated the benefits if the firms created an information-sharing alliance, and showed that if information sharing among allied firms had sufficiently large positive implications on firm requirements. The increased security information sharing can bring two benefits for the firms: a “direct benefit”, and a “strategic benefit” [43]. Considering two similar firms, the relationship between information sharing and information security investment is investigated. This research found that firms’ strategic choices vary with the features of stored information, either complementary or substitutable, and the investment strategy chosen by the firms might be sub-optimal [44].

Considering attacker behavior and leakage costs, the relationship between security investment and information sharing are further discussed. Their findings showed that firms should devote significant attention to their relationship with other firms when strategically choosing security investment [12]. By using differential game theoretic approaches, dynamic strategies for security investment and information sharing for two competing firms are investigated. This paper examined how security investment rates and information sharing rates are affected by several parameters in a non-cooperative scenario. Other similar studies have been conducted by [45,46,47]. We summarize the features of existing game theoretic approaches to security and privacy problems in Table 1.

As shown in Table 1, in previous works, most of game theoretic models of security and privacy investment are based on the assumption of a single scenario with an offender-defender interaction. An offender attempts to breach system security to disclose or cause damage to the privacy data. A defender, on the other hand, takes appropriate measures to enhance the level of privacy protection. However, the types of entities in a mHealth system might be multiple, and could include offenders, system providers, hospitals, patients and governments. The relationships between these entities are not only oppositional in nature. The relationships can also be cooperative, selfish, or free riding. Moreover, it is difficult to achieve the optimal investment strategy in a single game scenario where there is incomplete information and bounded rationality. Security and privacy investment studies based on other games, such as Bayesian, Stackelberg and differential games cannot solve this problem. Also, perfect rationality may not be practical in this scenario. Furthermore, existing studies seldom consider the impact of government regulation on investment. In the absence of appropriate regulation, the entities that invest in privacy protection (e.g., system providers and hospitals) will attempt to free ride on the privacy expenditures of others. How to motivate the entities to cooperate in privacy investment is not investigated in existing articles.

To distinguish this study from the models in existing works, we propose a parametric evolutionary game model. Our model consists of system providers, hospitals and governments, and can be used to analyze the optimal strategies of privacy investment in a mHealth system. Our evolutionary game theory (EGT) model which contains three types of entities, can potentially find several realistic stable strategies after recursive interactions. This study is designed to fill the gap in existing literature by exploring strategies of privacy investment. Our study also examines the impact of profit growth coefficients, investment costs, reputation profits and fines on the strategic choice of the participants

## 3. Evolutionary Game Model of Privacy Investment

### 3.1. Problem Recognition and Description

As well known, there is a large population in China, and the healthcare resources are in short supply [48]. As a typical mobile service, mHealth can provide care through telemedicine, reduce the costs, and improve the quality and timely access of healthcare. Therefore, the implementation of mHealth devices and applications has great significance and has risen to the national strategy of China [49]. However, privacy violation has become one of the most serious obstacles to the implementation of mHealth [50,51].

According to analysis above, an effective mechanism for mHealth privacy protection requires moderate investments of different entities, which may include technological research, devices purchase, organizational restructure, staff training, etc. Therefore, mHealth system providers, hospitals and governments have very important positions in mHealth privacy protection, while the factors that influence investors’ strategic choice should be deeply investigated. With these initial impressions, we went to Jiangsu Province Hospital of TCM, who has well-established mHealth systems in Nanjing, China and interviewed the hospital dean in charge of financial budgets, the manager in charge of mHealth service, several doctors and patients. In addition, we also went to iFLYTEK, a highly regarded mHealth system provider and interviewed the manager in charge of financial budgets and market, several system engineers and the relevant personnel of mHealth system. Through investigation, we found there were two outstanding problems of privacy investment:

(1) Insufficient budget is viewed as the main challenge for sustaining privacy investment because of the long return period and high investment costs.
(2) Because of information asymmetry and market dynamics, system providers and hospitals are not sure that privacy investment can provide competitive advantages. Therefore, they adjust their strategic choice frequently for profits maximization.

Concerning the first problem, the basic cost-benefit analysis is essential for both system providers and hospitals [52,53]. Economic factors, such as investment costs, profits from privacy investment and governmental subsidies will have significant impacts on the strategic choice of the above entities. Moreover, the investment process might create a channel that allows other entities to receive a free ride on privacy expenditures. Therefore, the interaction between game players will also influence their investment decisions. Considering these characteristics, game theory provides a quantitative decision framework that can formalize strategic interaction among autonomous players, and model privacy investment problems in which multiplayers with contradictory or collaborative goals [54,55]. Now, there has been substantial progress in the study of security and privacy investment based on game theoretic approaches [12,15,38,42].

However, concerning the second problem, system providers, hospitals and governments are bounded rationality because of incomplete information and dynamic investment process. It is difficult for investors to achieve an optimal strategy in a single game process by using Bayesian game, Stackelberg game and differential game theoretic approaches. Instead, they always adjust and improve their strategic choice for profits maximization. This characteristic is consistent with the evolution of nature, which motivate us to use evolutionary game theory.

Considering the decision problems of mHealth privacy investment, this paper applies evolutionary game theory to model such situations. We investigate the optimal strategies of privacy investment in mHealth context not only based on cost-benefit analysis, but also from evolutionary perspective. The motivation of using evolutionary game theory can be concluded as follows:

(1) Equilibrium solution refinement. The evolutionary game approaches provide a refined solution that ensures stability of a strategy adopted by a population, where no small subgroup of deviants could successfully invade the whole population. Such strategy is known as evolutionary stable strategy (ESS) [56].
(2) Bounded rationality. In traditional game theory, the game players are assumed as rational and the players believe that the other side is also rational throughout the game. However, this assumption is often unrealistic. This situation is avoided in evolutionary game, where players adopt dynamic strategies that lead them to sustain in the population without caring about instant profits maximization [57].
(3) Game dynamics. Since players in evolutionary game interact with each other for multiple rounds by adopting different strategies, the state of their interaction varies over time according to the replication games. Thus, the evolutionary game provides a natural way to introduce dynamics, where success strategies are imitated by others and propagate over interaction rounds [13].

### 3.2. Model Establishment

The privacy concerns of a mHealth system involve several types of entities, including offenders, defenders, patients and regulators. Their roles in privacy protection of mHealth system are described as follows:

(1) *Offenders.* Offenders maliciously use their computer/security skills to steal, exploit, and sell patient’s data. They always attempt to find and exploit weaknesses and vulnerabilities in security systems for illegal profit.
(2) *Defenders.* Defenders in a mHealth system include system providers and hospitals. System providers are technology providers (e.g., Microsoft, Amazon and Google) that can provide support to secure mHealth devices, databases and software for hospitals. Whether or not they will invest in privacy protection is dependent on the profits from and costs of those investments. Hospitals collect patient data from various sources, and perform other operations such as deleting, editing, and sharing data. The security level of patients’ data is positively related to the level of privacy management and the regulation of hospitals, which are also required to make sufficient investment.
(3) *Patients*. Patient’s data that is collected by mHealth devices will be incorporated into an electronic record that is stored in hospital databases. The electronic record is sought by and may potentially be shared with medical experts, caregivers, academic researchers and public health organizations [58]. To prevent privacy disclosure, we assume that patients are willing to pay more for improved service and privacy protection.
(4) *Regulators*. Regulators in a mHealth system refer to third-party organizations, such as governments. Under the current profit condition of privacy investment, governments should motivate system providers and hospitals to invest in privacy protection via punishment and compensation mechanisms.

**Figure 1:**
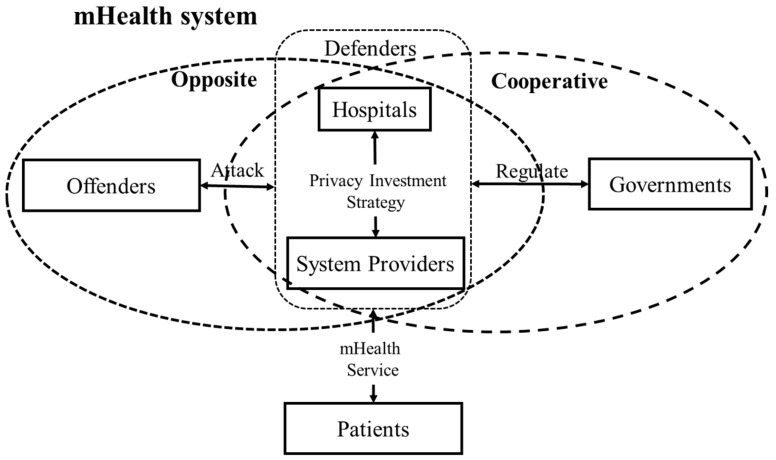
The relationship of entities in mHealth systems.

The relationship of the above entities is shown in Figure 1. Existing research primarily consider the relationship of participating entities to be opposite and competitive (e.g., offenders and defenders, offenders and patients). However, the relationship of entities in real life situations can be cooperative (e.g., hospitals and system providers) and reciprocal (e.g., hospitals and governments, system providers and governments), while the strategic choice can be affected by others.

This study proposes a mHealth system privacy investment chain, consisting of three types of entities: system providers (denoted by *S*), hospitals (denoted by *H*) and governments (denoted by G). System providers and hospitals have two types of strategies: “invest (I)” and “not invest (NI)”. The privacy investments of system providers include investment in technological research, software upgrades, hardware improvements, etc. The investment of hospitals includes equipment purchases, development of privacy rules, staff training, etc. We identify governments as key players because governments can charge fines to system providers and hospitals that choose to “not invest”. In current economic and technical conditions, governments can only charge static quantity-based fines to the entities that choose to “not invest”. Therefore, governments also have two strategies: “regulate (R)” and “not regulate (NR)”. Therefore, there are eight types of strategy profile between system providers, hospitals and governments: (invest, invest, regulate); (not invest, invest, regulate); (invest, not invest, regulate); (not invest, not invest, regulate); (invest, invest, not regulate); (not invest, invest, not regulate); (invest, not invest, not regulate); (not invest, not invest, not regulate).

#### 3.2.1. Notations

The key notations that occur in this paper are listed in Table 2 for easy reference.

#### 3.2.2. Assumptions and Payoff Matrix

Considering the reality of mHealth privacy investment in China, we posit the following assumptions to facilitate the model formulation and solution:

(1) With the rapid development of information and communication technology (ICT), personal health information in a digital format can be conveniently copied, transmitted, and integrated. Under this scenario, malicious offenders can easily use their professional skills to steal, exploit, and sell patient’s data for illegal benefits. According to our interview in Jiangsu Province Hospital of TCM and other hospitals, we find that patients are willing to pay more for the improved privacy protection of mHealth service for the reason of increased security/privacy incidents and the improvement of privacy awareness.
(2) Under current technical conditions, the investment cost is so high that the three types of game entities cannot make the optimal strategic decision at the initial stage. They have bounder rationality, and cannot make a choice that maximizes their own profits. Each player has imitating abilities and can adjust his or her own strategy according to experience.
(3) If system providers and hospitals simultaneously choose to “not invest”, patients will not pay more for a low level of privacy protection. The profits of system providers and hospitals remains fixed, which can be written, as *P_S_* and *P_H_*, respectively. In this scenario, if governments choose to “regulate”, system providers and hospitals will receive a fine *F*. Therefore, the payoff functions of game players can be defined as:
  (1) $$E_{S}(NI,NI,R)=P_{S}-F$$
  (2) $$E_{H}(NI,NI,R)=P_{H}-F$$
  (3) $$E_{G}(NI,NI,R)=2F-C_{G}$$
(4) If only system providers choose to “invest”, this will encourage technology research and software upgrades. Patient’s privacy can be protected at a higher level and patients are therefore willing to pay more. However, hospitals might free ride off the investment made by system providers and share in the extra benefits (such as having a positive reputation and earning patient trust). Thus, the hospitals will earn a larger profits *ξ_H_*. In this scenario, if governments choose to “regulate”, hospitals will receive the fine *F* and system providers will get the subsidy. Therefore, the payoff functions of entities can be defined as:
  (4) $$E_{S}(I,NI,R)=(1+\alpha_{0})P_{S}-C_{S}+F$$
  (5) $$E_{H}(I,NI,R)=\xi_{H}-F$$
  (6) $$E_{G}(I,NI,R)=R_{0}-C_{G}$$
(5) If only hospitals choose to “invest”, staff behavior can be regulated, and privacy awareness can be improved. Patients are also willing to pay more for effective privacy protection. Similarly, system providers also might share in the extra profits *ξ_S_* by free riding. In this scenario, if governments choose to “regulate”, then system providers will receive the fine *F* and hospitals will receive the subsidy. Therefore, the payoff functions of entities can be defined as:
  (7) $$E_{S}(NI,I,R)=\xi_{S}-F$$
  (8) $$E_{H}(NI,I,R)=(1+\beta_{0})P_{H}-C_{H}+F$$
  (9) $$E_{G}(NI,I,R)=R_{0}-C_{G}$$
(6) As mentioned above, patient’s privacy can be protected at a higher level even if only one side of the system providers and hospitals choose to “invest”. In this scenario, governments can obtain reputation profits *R*_0_ (*R*_0_ > 0) if they choose to “regulate”. However, if governments choose to “not regulate”, it will result in low efficiency of privacy protection and free riding might be present. Therefore, they will not obtain reputation profits.
(7) In practice, not all the behavior of privacy investment or free riding can be assessed precisely, for the sake of limited budgets and technological support. Therefore, we assume that the fine for system providers and hospitals is not enough, and smaller than reputation profits (2*F* < *R*_0_).
(8) If both the system providers and hospitals choose to “invest”, patient’s privacy can be protected more effectively, and patients will be willing to pay more. In this scenario, whether or not governments choose to “regulate”, they will receive higher reputation profits *R*_1_ (*R*_1_ > *R*_0_). Therefore, the payoff functions of entities can be defined as:
  (10) $$E_{S}(I,I,R)=(1+\alpha_{1})P_{S}-C_{S}$$
  (11) $$E_{H}(I,I,R)=(1+\beta_{1})P_{H}-C_{H}$$
  (12) $$E_{G}(I,I,R)=R_{1}-C_{G}$$

These assumptions are either common to most game theoretical approaches or closer to scenarios in reality. Based on the above assumptions, the payoff matrix for game players is shown in Table 3.

#### 3.2.3. Equilibrium Analysis

In the initial stage of the three types of players’ game, suppose that the population of system providers choosing “invest” is *x*(0 ≤ *x* ≤ 1), and the population choosing “not invest” is 1−*x*. Similarly, the population of hospitals choosing “invest” is *y*(0 ≤ *y* ≤ 1), and the population choosing “not invest” is 1−*y*. The population of governments choosing “regulate” is *z*(0 ≤ *z* ≤ 1), and the population choosing “not regulate” is 1−*z*.

According to the assumption in Section 3.2.2, supposing that *μ*_1,1_ represents the expected payoff of system providers that choose to “invest”, *μ*_1,2_ represents the expected payoff of system providers that choose “not invest”, and *μ*_1_ represents the average expected payoff. Then:

(13) $$\begin{array}{cc} \mu_{1,1} & =[(1+\alpha_{1})P_{S}-C_{S}]yz+[(1+\alpha_{0})P_{S}-C_{S}+F](1-y)z+[(1+\alpha_{1})P_{S}-C_{S}]y(1-z)\\ & +[(1+\alpha_{0})P_{S}-C_{S}](1-y)(1-z)\\ & =(1+\alpha_{0})P_{S}-C_{S}+(\alpha_{1}-\alpha_{0})P_{S}y+F(1-y)z \end{array}$$

(14) $$\begin{array}{cc} \mu_{1,2} & =(\xi_{S}-F)yz+(P_{S}-F)(1-y)z+\xi_{S}y(1-z)+P_{S}(1-y)(1-z)\\ & =(\xi_{S}-P_{S})y+P_{S}-Fz \end{array}$$

(15) $$\mu_{1}=x\mu_{1,1}+(1-x)\mu_{1,2}$$

Supposing that *μ*_2,1_ represents the expected payoff of hospitals that choose to “invest”, *μ*_2,2_ represents the expected payoff of hospitals that choose to “not invest”, and *μ*_2_ represents the average expected payoff. Then:

(16) $$\begin{array}{cc} \mu_{2,1} & =[(1+\beta_{1})P_{H}-C_{H}]xz+[(1+\beta_{0})P_{H}-C_{H}+F](1-x)z+[(1+\beta_{1})P_{H}-C_{H}]x(1-z)\\ & +[(1+\beta_{0})P_{H}-C_{H}](1-x)(1-z)\\ & =(1+\beta_{0})P_{H}-C_{H}+(\beta_{1}-\beta_{0})P_{H}x+F(1-x)z \end{array}$$

(17) $$\begin{array}{cc} \mu_{2,2} & =(\xi_{H}-F)xz+(P_{H}-F)(1-x)z+\xi_{H}x(1-z)+P_{H}(1-x)(1-z)\\ & =(\xi_{H}-P_{H})x+P_{H}-Fz \end{array}$$

(18) $$\mu_{2}=y\mu_{2,1}+(1-y)\mu_{2,2}$$

Supposing that *μ*_3,1_ represents the expected payoff of governments that choose to “regulate”, *μ*_3,2_ represents the expected payoff of governments that choose to “not regulate”, and *μ*_3_ represents the average expected payoff. Then:

(19) $$\begin{array}{cc} \mu_{3,1} & =(R_{1}-C_{G})xy+(R_{0}-C_{G})(1-x)y+(R_{0}-C_{G})x(1-y)+(2F-C_{G})(1-x)(1-y)\\ & =R_{1}xy+R_{0}[x(1-y)+(1-x)y]+2F(1-x)(1-y)-C_{G} \end{array}$$

(20) $$\begin{array}{cc} \mu_{3,2} & =R_{1}xy-L(1-x)y-Lx(1-y)-L(1-x)(1-y)\\ & =R_{1}xy-L+Lxy \end{array}$$

(21) $$\mu_{3}=z\mu_{3,1}+(1-z)\mu_{3,2}$$

According to the Malthusian dynamic equation [59], the replication dynamic equation of the population *x* for system providers is:

(22) $$\begin{array}{cc} F_{S}(x) & =\frac{dx}{dt}=x(\mu_{1,1}-\mu_{1})\\ & =x(1-x)[(\alpha_{0}P_{S}-C_{S})+(\alpha_{1}-\alpha_{0})P_{S}y-(\xi_{S}-P_{S})y+F(2-y)z] \end{array}$$

The replication dynamic equation of the population *y* for hospitals is:

(23) $$\begin{array}{cc} F_{H}(y) & =\frac{dy}{dt}=y(\mu_{2,1}-\mu_{2})\\ & =y(1-y)[(\beta_{0}B_{H}-C_{H})+(\beta_{1}-\beta_{0})B_{H}x-(\xi_{H}-B_{H})x+F(2-x)z] \end{array}$$

The replication dynamic equation of the population *z* for governments is:

(24) $$\begin{array}{cc} F_{G}(z) & =\frac{dz}{dt}=z(\mu_{3,1}-\mu_{3})\\ & =z(1-z)\{R_{0}[x(1-y)+(1-x)y]+2F(1-x)(1-y)-C_{G}+L-Lxy\} \end{array}$$

From the replication dynamic equations above, we have nine equilibrium points—*P*_1_(0,0,0), *P*_2_(0,0,1), *P*_3_(0,1,0), *P*_4_(0,1,1), *P*_5_(1,0,0), *P*_6_(1,0,1), *P*_7_(1,1,0), *P*_8_(1,1,1), *P*_9_(x*,y*,z*)—that correspond to equilibria of the dynamic system. *P*_9_(x*,y*,z*) is a mixed equilibrium point that satisfies the condition:

(25) $$\left\{ \begin{array}{l} (\alpha_{0}P_{S}-C_{S})+(\alpha_{1}-\alpha_{0})P_{S}y*-(\xi_{S}-P_{S})y*+F(2-y*)z*=0\\ (\beta_{0}B_{H}-C_{H})+(\beta_{1}-\beta_{0})B_{H}x*-(\xi_{H}-B_{H})x*+F(2-x*)z*=0\\ R_{0}[x*(1-y*)+(1-x*)y*]-C_{G}+L-Lx*y*=0 \end{array}\right.$$

### 3.3. Stable Analysis of Equilibrium Points

The stability of equilibrium points can be analyzed by using a Jacobian matrix [60]. The Jacobian matrix can be defined as follows:

(26) $$J=\left[\begin{array}{ccc} \frac{\partial F_{S}(x)}{\partial x} & \frac{\partial F_{S}(x)}{\partial y} & \frac{\partial F_{S}(x)}{\partial z}\\ \frac{\partial F_{H}(y)}{\partial x} & \frac{\partial F_{H}(y)}{\partial x} & \frac{\partial F_{H}(y)}{\partial z}\\ \frac{\partial F_{A}(z)}{\partial x} & \frac{\partial F_{A}(z)}{\partial y} & \frac{\partial F_{A}(z)}{\partial z} \end{array}\right]=\left[\begin{array}{ccc} a_{11} & a_{12} & a_{13}\\ a_{21} & a_{22} & a_{23}\\ a_{31} & a_{32} & a_{33} \end{array}\right]$$

We can examine the stability of equilibrium points by following the conditions *a*_11_ < 0, *a*_22_ < 0 and *a*_33_ < 0 [61]. Then, we can compute the values of the equilibrium points shown in Table 4. Here, *P*_9_ is not satisfied under the above condition because *a*_11_ = 0, *a*_22_ = 0 and *a*_33_ = 0. Here, *P*_8_(1,1,1) is not a satisfied condition because *C_G_* > 0. Other equilibrium points will be ESSs whereas the values of related parameters are satisfied under different conditions.

**Figure 2:**
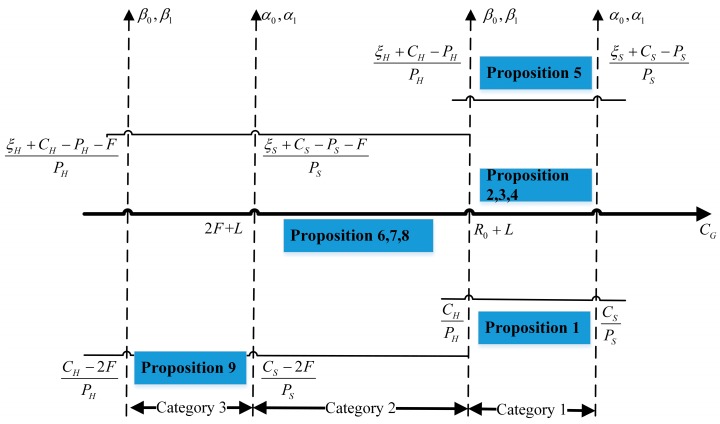
ESSs in different intervals.

According to Table 4, the ESSs of three players are correlated with regulation costs, regulation profits, payoff growth coefficients, and fine for investors. Therefore, the stability analysis of an equilibrium strategy can be categorized when those parameters are in different intervals. The schematic diagram is shown in Figure 2. To facilitate the analysis of ESSs, we assume that there is just a single government regulating all the system providers and hospitals available in a local area.

#### 3.3.1. ESSs When *C_G_* > *R*_0_ + *L*

If the regulation cost is high enough to satisfy the condition of *C_G_* > *R*_0_ + *L*, governments will choose to “not regulate”, whether or not system providers and hospitals choose to “invest”. The ESSs are elaborated by the following propositions:

**Proposition** **1.** *When $0<\alpha_{0}<\frac{C_{S}}{P_{S}}$, $\alpha_{0}<\alpha_{1}<\frac{\xi_{S}+C_{S}-P_{S}}{P_{S}}$ and $0<\beta_{0}<\frac{C_{H}}{P_{H}}$, $\beta_{0}<\beta_{1}<\frac{\xi_{H}+C_{H}-P_{H}}{P_{H}}$, (0, 0, 0) is an ESS, and then system providers, hospitals and governments will choose to (not invest, not invest, not regulate).*

**Proof.**  If $0<\alpha_{0}<\frac{C_{S}}{B_{S}}$, $0<\beta_{0}<\frac{C_{H}}{B_{H}}$ and $C_{G}>R_{0}+L>2F+L$, we find that:

$$E_{G}(N,N,R)=2F-C_{G}<-L=E_{G}(N,N,NR)$$

$$E_{S}(I,NI,NR)=(1+\alpha_{0})P_{S}-C_{S}<(1+\frac{C_{S}}{P_{S}})P_{S}-C_{S}=P_{S}=E_{S}(NI,NI,NR)$$

$$E_{H}(NI,I,NR)=(1+\beta_{0})P_{H}-C_{H}<(1+\frac{C_{H}}{P_{H}})P_{H}-C_{H}=P_{H}=E_{H}(NI,NI,NR)$$

In this scenario, the regulation cost is higher than the credit loss. Therefore, governments will choose to “not regulate”. Meanwhile, the profit growth coefficients are small, so system providers and hospitals have little or no incentive to invest in privacy protection (for small profits). Therefore, the ESS profile is to (not invest, not invest, not regulate). □

**Proposition** **2.** *When $0<\alpha_{0}<\frac{C_{S}}{P_{S}}$, $\alpha_{0}<\alpha_{1}<\frac{\xi_{S}+C_{S}-P_{S}}{P_{S}}$ and $\frac{C_{H}}{P_{H}}<\beta_{0}<\beta_{1}<\frac{\xi_{H}+C_{H}-P_{H}}{P_{H}}$, (0, 1, 0) is an ESS, and then system providers, hospitals and governments will prefer to (not invest, invest, not regulate).*

**Proof.**  If $C_{G}>R_{0}+L$, we find that $E_{G}(NI,I,R)=R_{0}-C_{G}<E_{G}(NI,I,NR)$. Therefore, governments will choose to “not regulate”. Then, if $\frac{C_{H}}{P_{H}}<\beta_{0}<\beta_{1}<\frac{\xi_{H}+C_{H}-P_{H}}{P_{H}}$, we find that:

$$E_{H}(NI,I,NR)=(1+\beta_{0})P_{H}-C_{H}>(1+\frac{C_{H}}{P_{H}})P_{H}-C_{H}=P_{H}=E_{H}(NI,NI,NR)$$

In this scenario, hospitals have a stronger incentive to invest in privacy protection. Then, if the profit growth coefficients of the system providers remain fixed, we can prove that

$$E_{S}(I,I,NR)=(1+\alpha_{1})P_{S}-C_{S}<(1+\frac{\xi_{S}+C_{S}-P_{S}}{P_{S}})P_{S}-C_{S}=\xi_{S}=E_{S}(NI,I,NR)$$

System providers prefer to choose to “not invest” because the profit from choosing “invest” is lower than the profit from free riding. Therefore, the ESS profile is to (not invest, invest, not regulate). □

**Proposition** **3.** *When $\frac{C_{S}}{P_{S}}<\alpha_{0}<\alpha_{1}<\frac{\xi_{S}+C_{S}-P_{S}}{P_{S}}$, $0<\beta_{0}<\frac{C_{H}}{P_{H}}$ and $\beta_{0}<\beta_{1}<\frac{\xi_{H}+C_{H}-P_{H}}{P_{H}}$, (1, 0, 0) is an ESS, and then system providers, hospitals and governments will choose to (invest, not invest, not regulate).*

**Proof.**  Similarly, we find that $E_{G}(I,NI,R)=R_{0}-C_{G}<E_{G}(I,NI,NR)$. Therefore, governments will choose to “not regulate”. Then, as the profit growth coefficients of system providers increase, which are satisfied by $\frac{C_{S}}{P_{S}}<\alpha_{0}<\alpha_{1}<\frac{\xi_{S}+C_{S}-P_{S}}{P_{S}}$, we find that:

$$E_{S}(I,NI,NR)=(1+\alpha_{0})P_{S}-C_{S}>(1+\frac{C_{S}}{P_{S}})P_{S}-C_{S}=P_{S}=E_{S}(NI,NI,NR)$$

In this scenario, system providers will invest in privacy protection. We can also prove that

$$E_{H}(I,I,NR)=(1+\beta_{1})P_{H}-C_{H}<(1+\frac{\xi_{H}+C_{H}-P_{H}}{P_{H}})P_{H}-C_{H}=\xi_{H}=E_{H}(I,NI,NR)$$

Hospitals prefer to “not invest” because the profit from choosing to “invest” is lower than the profit from free riding. The ESS is to (invest, not invest, not regulate). □

**Proposition** **4.** *When $\frac{C_{S}}{P_{S}}<\alpha_{0}<\alpha_{1}<\frac{\xi_{S}+C_{S}-P_{S}}{P_{S}}$ and $\frac{C_{H}}{P_{H}}<\beta_{0}<\beta_{1}<\frac{\xi_{H}+C_{H}-P_{H}}{P_{H}}$, (1, 0, 0) and (0, 1, 0) are ESSs, then system providers, hospitals and governments will choose to (invest, not invest, not regulate) or to (not invest, invest, not regulate).*

**Proof.**  Similarly, we know that governments will choose to “not regulate”. Then, if $\frac{C_{S}}{P_{S}}<\alpha_{0}<\alpha_{1}<\frac{\xi_{S}+C_{S}-P_{S}}{P_{S}}$ and $\frac{C_{H}}{P_{H}}<\beta_{0}<\beta_{1}<\frac{\xi_{H}+C_{H}-P_{H}}{P_{H}}$, we find that:

$$E_{S}(I,NI,NR)=(1+\alpha_{0})P_{S}-C_{S}>(1+\frac{C_{S}}{P_{S}})P_{S}-C_{S}=P_{S}=E_{S}(NI,NI,NR)$$

$$E_{H}(NI,I,NR)=(1+\beta_{0})P_{H}-C_{H}>(1+\frac{C_{H}}{P_{H}})P_{H}-C_{H}=P_{H}=E_{H}(NI,NI,NR)$$

$$E_{S}(I,I,NR)=(1+\alpha_{1})P_{S}-C_{S}<(1+\frac{\xi_{S}+C_{S}-P_{S}}{P_{S}})P_{S}-C_{S}=\xi_{S}=E_{S}(NI,I,NR)$$

$$E_{H}(I,I,NR)=(1+\beta_{1})P_{H}-C_{H}<(1+\frac{\xi_{H}+C_{H}-P_{H}}{P_{H}})P_{H}-C_{H}=\xi_{H}=E_{H}(I,NI,NR)$$

The profits to system providers and hospitals that choose to “invest” are higher than the investment costs. However, the profits are lower than the profits from free riding. These entities may therefore free ride others, and the ESS profile can be to (invest, not invest, not regulate) or to (not invest, invest, not regulate) which depending on the initial stage. □

**Proposition** **5.** *When $\frac{\xi_{S}+C_{S}-P_{S}}{P_{S}}<\alpha_{0}<\alpha_{1}$ and $\frac{\xi_{H}+C_{H}-P_{H}}{P_{H}}<\beta_{0}<\beta_{1}$, (1, 1, 0) is an ESS, then system providers, hospitals and governments will choose to (invest, invest, not regulate).*

**Proof.**  If $\frac{\xi_{S}+C_{S}-P_{S}}{P_{S}}<\alpha_{0}<\alpha_{1}$ and $\frac{\xi_{H}+C_{H}-P_{H}}{P_{H}}<\beta_{0}<\beta_{1}$, we find that:

$$E_{S}(I,I,NR)=(1+\alpha_{1})P_{S}-C_{S}>(1+\frac{\xi_{S}+C_{S}-P_{S}}{P_{S}})P_{S}-C_{S}=\xi_{S}=E_{S}(NI,I,NR)$$

$$E_{H}(I,I,NR)=(1+\beta_{1})P_{H}-C_{H}>(1+\frac{\xi_{H}+C_{H}-P_{H}}{P_{H}})P_{H}-C_{H}=\xi_{H}=E_{H}(I,NI,NR)$$

In this scenario, the profits for system providers and hospitals that choose to “invest” are higher than both the investment costs and the profits from free riding. Both of these entities will therefore choose to “invest”, even without the imposition of regulations. Therefore, the ESS profile is to (invest, invest, not regulate). □

#### 3.3.2. ESSs When 2*F* + *L* < *C_G_* < *R*_0_ + *L*

If the regulation cost is satisfied by the condition of 2*F* + *L* < *C_G_* < *R*_0_ + *L* , then governments will choose to “regulate” when system providers and/or hospitals choose to “invest”. The entities will receive a fine if they do not choose to “invest”. Here, ESSs are elaborated by the following propositions:

**Proposition** **6.** *When $0<\alpha_{0}<\frac{C_{S}-2F}{P_{S}}$, $\alpha_{0}<\alpha_{1}<\frac{\xi_{S}+C_{S}-P_{S}-F}{P_{S}}$ and $\frac{C_{H}-2F}{P_{H}}<\beta_{0}<\beta_{1}<\frac{\xi_{H}+C_{H}-P_{H}-F}{P_{H}}$, (0, 1, 1) is an ESS, then system providers, hospitals and governments will choose to (not invest, invest, regulate).*

**Proof.**  Because $C_{G}<R_{0}+L$, we find that $E_{G}(NI,I,R)=R_{0}-C_{G}>-L=E_{G}(NI,I,NR)$. Therefore, governments will choose to “regulate”. Then, if $\frac{C_{H}-2F}{P_{H}}<\beta_{0}<\beta_{1}<\frac{\xi_{H}+C_{H}-P_{H}-F}{P_{H}}$, we find that:

$$E_{H}(NI,I,R)=(1+\beta_{0})P_{H}-C_{H}+F>(1+\frac{C_{H}-2F}{P_{H}})P_{H}-C_{H}=P_{H}-F=E_{H}(NI,NI,R)$$

Therefore, hospitals will invest in privacy protection when they can obtain the subsidy F. If $0<\alpha_{0}<\frac{C_{S}-2F}{P_{S}}$ and $\alpha_{0}<\alpha_{1}<\frac{\xi_{S}+C_{S}-P_{S}}{P_{S}}$, we can prove that:

$$E_{S}(I,I,R)=(1+\alpha_{1})P_{S}-C_{S}<(1+\frac{\xi_{S}+C_{S}-P_{S}-F}{P_{S}})P_{S}-C_{S}=\xi_{S}-F=E_{S}(NI,I,R)$$

In this scenario, the profit for system providers choosing to “invest” is lower than the profit from free riding even if the system providers may receive the fine F. Therefore, the ESS profile is to (not invest, invest, regulate). □

**Proposition** **7.** *When $\frac{C_{S}-2F}{P_{S}}<\alpha_{0}<\alpha_{1}<\frac{\xi_{S}+C_{S}-P_{S}-F}{P_{S}}$, $0<\beta_{0}<\frac{C_{H}-2F}{P_{H}}$ and $\beta_{0}<\beta_{1}<\frac{\xi_{H}+C_{H}-P_{H}-F}{P_{H}}$, (1, 0, 1) is an ESS, then system providers, hospitals and governments will choose to (invest, not invest, regulate).*

**Proof.**  If $\frac{C_{S}-2F}{P_{S}}<\alpha_{0}<\alpha_{1}<\frac{\xi_{S}+C_{S}-P_{S}-F}{P_{S}}$, we find that:

$$E_{S}(I,NI,R)=(1+\alpha_{0})P_{S}-C_{S}+F>(1+\frac{C_{S}-2F}{P_{S}})P_{S}-C_{S}=P_{S}-F=E_{S}(NI,NI,R)$$

Therefore, system providers will choose to “invest” when they can obtain the subsidy F. Then, if $0<\beta_{0}<\frac{C_{H}-2F}{P_{H}}$ and $\beta_{0}<\beta_{1}<\frac{\xi_{H}+C_{H}-P_{H}-F}{P_{H}}$, we can prove that:

$$E_{H}(I,I,R)=(1+\beta_{1})P_{H}-C_{H}<(1+\frac{\xi_{H}+C_{H}-P_{H}-F}{P_{H}})P_{H}-C_{H}=\xi_{H}-F=E_{H}(I,NI,R)$$

The profit for hospitals choosing to “invest” is lower than the profit from free riding even if they receive the fine F. Therefore, the ESS profile is to (invest, not invest, regulate). □

**Proposition** **8.** *When $\frac{C_{S}-2F}{P_{S}}<\alpha_{0}<\alpha_{1}<\frac{\xi_{S}+C_{S}-P_{S}-F}{P_{S}}$ and $\frac{C_{H}-2F}{P_{H}}<\beta_{0}<\beta_{1}<\frac{\xi_{H}+C_{H}-P_{H}-F}{P_{H}}$, (1, 0, 1) and (0, 1, 1) are ESSs, then system providers, hospitals and governments will choose to (invest, not invest, regulate) or to (not invest, invest, regulate).*

**Proof.**  If $\frac{C_{S}-2F}{P_{S}}<\alpha_{0}<\alpha_{1}<\frac{\xi_{S}+C_{S}-P_{S}-F}{P_{S}}$ and $\frac{C_{H}-2F}{P_{H}}<\beta_{0}<\beta_{1}<\frac{\xi_{H}+C_{H}-P_{H}-F}{P_{H}}$ , we find:

$$E_{S}(I,NI,R)=(1+\alpha_{0})P_{S}-C_{S}+F>(1+\frac{C_{S}-2F}{P_{S}})P_{S}-C_{S}=P_{S}-F=E_{S}(NI,NI,R)$$

$$E_{H}(NI,I,R)=(1+\beta_{0})P_{H}-C_{H}+F>(1+\frac{C_{H}-2F}{P_{H}})P_{H}-C_{H}=P_{H}-F=E_{H}(NI,NI,R)$$

$$E_{S}(I,I,R)=(1+\alpha_{1})P_{S}-C_{S}<(1+\frac{\xi_{S}+C_{S}-P_{S}-F}{P_{S}})P_{S}-C_{S}=\xi_{S}-F=E_{S}(NI,I,R)$$

$$E_{H}(I,I,R)=(1+\beta_{1})P_{H}-C_{H}<(1+\frac{\xi_{H}+C_{H}-P_{H}-F}{P_{H}})P_{H}-C_{H}=\xi_{H}-F=E_{H}(I,NI,R)$$

The profits for system providers and hospitals that choose to “invest” are higher than the investment costs, but the profits are lower than the profits from free riding. These entities may therefore free ride others, even when they might be faced with the fine F by governments. Therefore, the ESS can be either to (invest, not invest, regulate) or to (not invest, invest, regulate). □

#### 3.3.3. ESSs When *C_G_* < 2*R* + *L*

If the regulation cost is low and therefore satisfied the condition of *C_G_* < 2*R* + *L*, then governments will choose to “regulate” even if they will not gain the desired reputation profits. The ESS is illustrated as follows.

**Proposition** **9.** *When $0<\alpha_{0}<\frac{C_{S}-2F}{P_{S}}$ and $0<\beta_{0}<\frac{C_{H}-2F}{P_{H}}$, (0, 0, 1) is an ESS and then, system providers, hospitals and governments will choose to (not invest, not invest, regulate).*

**Proof.**  Because $C_{G}<2F+L$, we know that $E_{G}(NI,NI,R)=2F-C_{G}>L=E_{G}(NI,NI,NR)$. Therefore, governments will choose to “regulate”. Then, if $0<\alpha_{0}<\frac{C_{S}-2F}{P_{S}}$ and $0<\beta_{0}<\frac{C_{H}-2F}{P_{H}}$, we can prove that:

$$E_{S}(I,NI,R)=(1+\alpha_{0})P_{S}-C_{S}+F<(1+\frac{C_{S}-2F}{P_{S}})P_{S}-C_{S}+F=P_{S}-F=E_{S}(NI,NI,R)$$

$$E_{H}(NI,I,R)=(1+\beta_{0})P_{H}-C_{H}+F<(1+\frac{C_{H}-2F}{P_{H}})P_{H}-C_{H}+F=P_{H}-F=E_{H}(NI,NI,R)$$

In this scenario, the profit growth coefficients are small. As such, system providers and hospitals will choose to “not invest” (because of the small profits), even if they can obtain the subsidy. Therefore, the ESS profile is to (not invest, not invest, regulate). □

## 4. Illustration and Simulation

### 4.1. Numerical Example

Our game equilibria provide a detailed exposition of the game model and its properties. In this section, we derive numerical results from the game analysis, and use MATLAB to simulate and support the game-theoretic analysis. The variables used to calculate the ESSs were $P_{S}$, $P_{H}$, $\xi_{S}$, $\xi_{H}$, $C_{S}$, $C_{H}$, $\alpha_{0}$, $\alpha_{1}$, $\beta_{0}$, $\beta_{1}$, F, $C_{G}$,$R_{0}$ and L. We assign fixed values to several variables, whereas other variables will increase or decrease related to assigned variables. Please note that the values we used in this MATLAB simulation are just for illustration. In reality, the values of these parameters are determined by the financial earnings, investment costs, and the quantify measurement of reputation.

According to the analysis in Section 4, ESSs are different when the regulation costs and the profit growth coefficients occur under different conditions. Therefore, the numerical simulation can be analyzed under different values of $C_{G}$, $R_{0}$, F and L, as shown in Table 5.

### 4.2. Simulation of ESSs

#### 4.2.1. Simulation When *C_G_* > 2*R* + *L*

We set the values of the included parameters to $C_{G}$ = $400, $R_{0}$ = $200, L = $100, $P_{S}$ = $500, $P_{H}$ = $400, $\xi_{S}$ = $700, $\xi_{H}$ = $600, $C_{S}$ = $200, $C_{H}$ = $100. Also, $\alpha_{0}$, $\alpha_{1}$, $\beta_{0}$ and $\beta_{1}$ are variables. Thus, we can calculate the following:

$$\frac{C_{S}}{P_{S}}=0.4,\;\frac{C_{H}}{P_{H}}=0.25,\;\frac{\xi_{S}+C_{S}-P_{S}}{P_{S}}=0.8\;\mathrm{and}\;\frac{\xi_{H}+C_{H}-P_{H}}{P_{H}}=0.75$$

Governments will choose to “not regulate” because *C_G_* < 2*R* + *L*. Therefore, the replication dynamic equation of population *z* for governments can be defined as *z* = 0. Then, we set the replication dynamic equation of population *x*,*y* for system providers and hospitals to be from 5% to 95%.

**Figure 3:**
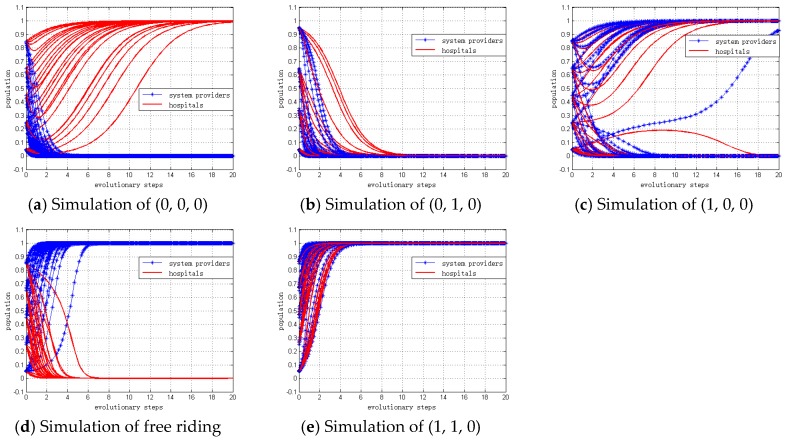
Simulation when $C_{G}>R_{0}+L$.

Therefore, the numerical simulation of different ESSs can be analyzed under the different values of $\alpha_{0}$, $\alpha_{1}$, $\beta_{0}$ and $\beta_{1}$, as shown in Table 6. The simulation results are depicted in Figure 3, which are consistent with the theoretical analyses of Proposition 1 to Proposition 5.

#### 4.2.2. Simulation When 2*F* + *L* < *C_G_* < *R*_0_ + *L*

When 2*F* + *L* < *C_G_* < *R*_0_ + *L* , governments will choose to “regulate” if any of the system providers and/or hospitals choose to “invest”. Therefore, the replication dynamic equation of population z for governments can be defined as *z* = 1. Similarly, we set the replication dynamic equation of population x,y for system providers and hospitals to be from 5% to 95%.

To facilitate our simulation, we set the values of included parameters to $C_{G}$ = $250, $R_{0}$ = $200, L = $100, F = $40, $P_{S}$ = $500, $P_{H}$ = $400, $\xi_{S}$ = $700, $\xi_{H}$ = $600, $C_{S}$ = $200, $C_{H}$ = $150. Also, $\alpha_{0}$, $\alpha_{1}$, $\beta_{0}$ and $\beta_{1}$ are variables. Thus, we can calculate the following:

$$\frac{C_{S}-2F}{P_{S}}=0.24,\;\frac{C_{H}-2F}{P_{H}}=0.175,\;\frac{\xi_{S}+C_{S}-P_{S}-F}{P_{S}}=0.72\;\mathrm{and}\;\frac{\xi_{H}+C_{H}-P_{H}-F}{P_{H}}=0.775$$

**Figure 4:**
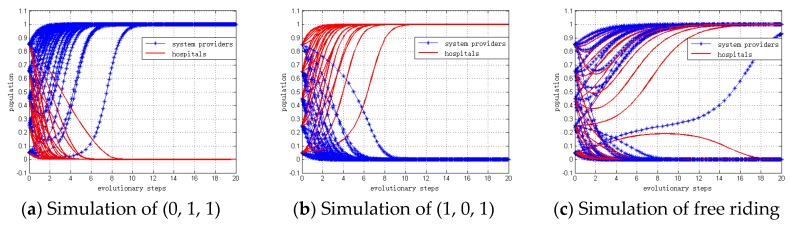
Simulation when $2F+L<C_{G}<R_{0}+L$.

Therefore, the numerical simulation of different ESSs can be analyzed under different values of $\alpha_{0}$, $\alpha_{1}$, $\beta_{0}$ and $\beta_{1}$ as shown in Table 7. The simulation results are depicted in Figure 4, and these results support Proposition 6 to Proposition 8.

#### 4.2.3. Simulation When *C_G_* < 2*F* + *L*

When *C_G_* < 2*F* + *L*, governments will always prefer to “regulate”. Therefore, the replication dynamic equation of population *z* for governments can be defined as *z* = 1. The replication dynamic equation of population x,y for system providers and hospitals is also set at from 5% to 95%.

We set the values of the included parameters to $C_{G}$ = $80, $R_{0}$ = $200, L = $100, F = $40, $P_{S}$ = $500, $P_{H}$ = $400, $\xi_{S}$ = $700, $\xi_{H}$ = $600, $C_{S}$ = $200, $C_{H}$ = $150. Also, $\alpha_{0}$, $\alpha_{1}$, $\beta_{0}$ and $\beta_{1}$ are variables. Thus, we can calculate the following:

$$\frac{C_{S}-2F}{P_{S}}=0.24,\;\frac{C_{H}-2F}{P_{H}}=0.175,\;\frac{\xi_{S}+C_{S}-P_{S}-F}{P_{S}}=0.72\;\mathrm{and}\;\frac{\xi_{H}+C_{H}-P_{H}-F}{P_{H}}=0.775$$

We set $\alpha_{0}$ = 0.2, $\alpha_{1}$ = 0.6, $\beta_{1}$ =0.1, $\beta_{1}$ = 0.3, which satisfies the following condition:

$$0<\alpha_{0}<\frac{C_{S}-2F}{P_{S}}\;\mathrm{and}\;0<\beta_{0}<\frac{C_{H}-2F}{P_{H}}$$

**Figure 5:**
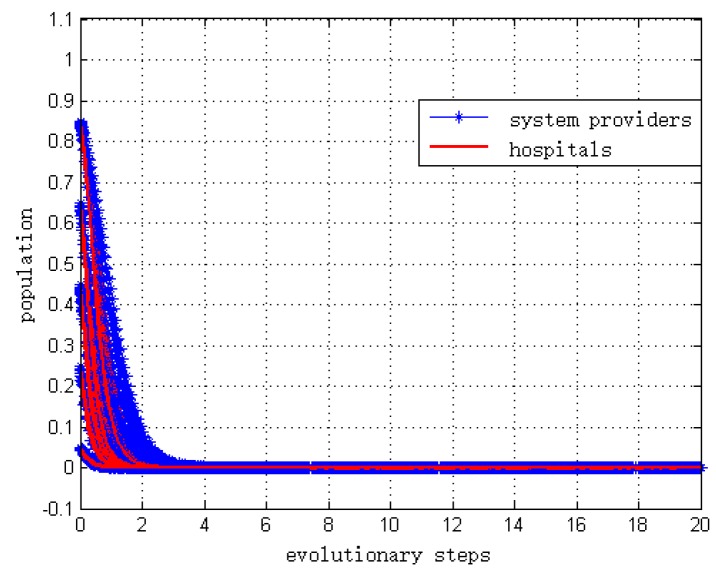
Simulation when $C_{G}<2F+L$.

The simulation result is shown in Figure 5. This result is consistent with the theoretical analysis of Proposition 9.

### 4.3. Sensitivity Analysis and Discussion

Next, we conduct a sensitivity analysis to explain the impact of $\alpha_{0}$, $\alpha_{1}$, $\beta_{0}$, $\beta_{1}$ and F on ESSs. We take stable points (0, 0, 0), (0, 1, 1) and (0, 0, 1) as examples, and other scenarios are similar.

#### 4.3.1. Sensitivity Analysis of (0, 0, 0)

First, we perform a sensitivity analysis of (0, 0, 0). The values of $\alpha_{0}$, $\alpha_{1}$, $\beta_{0}$ and $\beta_{1}$ vary within a fixed range, as are summarized in Table 8.

Then, we set the initial population *x*,*y* as follows:

x=0.2,y=0.8; x=0.3,y=0.7; x=0.4,y=0.6

**Figure 6:**
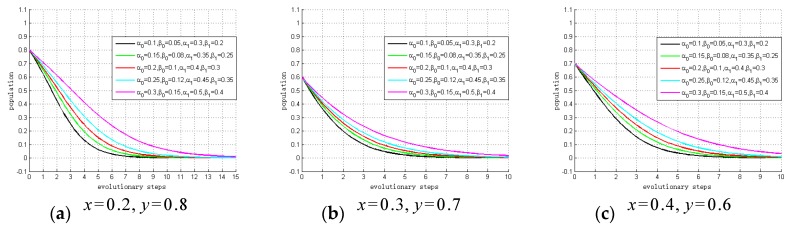
Sensitivity analysis of (0, 0, 0).

Figure 6 summarizes the results of the sensitivity analysis. The results show the relationship between profit growth coefficients and the evolutionary trend. As shown, the smaller the profit growth coefficients (*α*_0_, *α*_1_, *β*_0_, *β*_1_, $\alpha_{1}$, $\beta_{0}$, $\beta_{1}$) are, the fewer steps there are to ESS. In other words, the lower the profit brought about by privacy investment, the larger becomes the probability of choosing to “not invest”. Hence, if governments choose to “not regulate”, system providers and hospitals will tend to choose “not invest” due to the small profit growth coefficients. The simulation results of our sensitivity analysis support Proposition 1, and Proposition 2 to Proposition 5 can be verified by similar methods.

#### 4.3.2. Sensitivity Analysis of (0, 1, 1)

When governments choose to “regulate”, the entity that chooses to “not invest” will receive the fine F. Conversely, the entity that chooses to ‘invest’ will get the subsidy F. Therefore, we conduct a sensitivity analysis to explain the impact of F on ESS. The values of F vary within a fixed range that can be defined as follows:

F=30; F=35; F=40

Then, we set the initial population x,y as follows:

x=0.2,y=0.8; x=0.3,y=0.7; x=0.4,y=0.6

**Figure 7:**
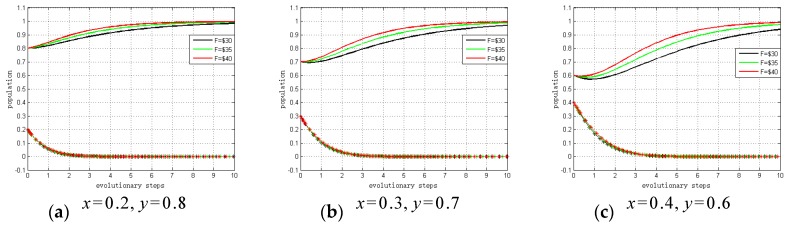
Sensitivity analysis of stable point (0, 1, 1).

Figure 7 summarizes the sensitivity analysis results. The results show the relationship between fines/subsidies and evolutionary trends. As shown, the larger the fine/subsidy (F) is, the fewer steps there are to ESS. Hence, if governments choose to “regulate”, there is a far greater probability that system providers and hospitals will choose to “invest” due to the potential fine or subsidy. That is, if the profit growth coefficients are satisfied $0<\alpha_{0}<\frac{C_{S}-2F}{P_{S}}$, $\alpha_{0}<\alpha_{1}<\frac{\xi_{S}+C_{S}-P_{S}-F}{P_{S}}$ and $\frac{C_{H}-2F}{P_{H}}<\beta_{0}<\beta_{1}<\frac{\xi_{H}+C_{H}-P_{H}-F}{P_{H}}$, the conditions will exist whereby either system providers or hospitals will invest in privacy protection. These results support Proposition 6, and Proposition 7 and Proposition 8 can be verified by similar methods.

#### 4.3.3. Sensitivity Analysis of (0, 0, 1)

If none of the system providers and hospitals chooses to “invest”, both of them will receive the fine F. Therefore, we conduct a sensitivity analysis to explain the impact of *F* on ESS. The values of *F* vary within a fixed range that can be defined as follows: F=30; F=35; F=40.

Then, we set the initial population x,y as follows:

x=0.2,y=0.8; x=0.3,y=0.7; x=0.4,y=0.6

**Figure 8:**
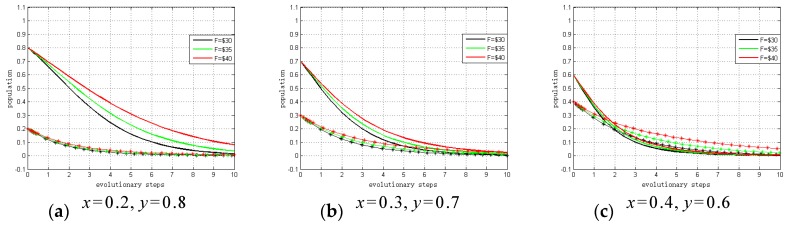
Sensitivity analysis of stable point (0, 0, 1).

Figure 8 summarizes the sensitivity analysis results, which show the relationship between fines and evolutionary trends. As shown, the smaller the fine/subsidy (*F*) is, the fewer steps there are to ESS. Hence, under these circumstances system providers and hospitals will tend to choose to “not invest” due to the small profit growth coefficients, even when they know will receive the fine. That is, if the profit growth coefficients are satisfied $0<\alpha_{0}<\frac{C_{S}-2F}{P_{S}}$ and $0<\beta_{0}<\frac{C_{H}-2F}{P_{H}}$, the conditions will exist whereby none of system providers and hospitals will invest in privacy protection. These sensitivity analysis results support Proposition 9.

### 4.4. Impacts of Different Parameters on ESS

**Figure 9:**
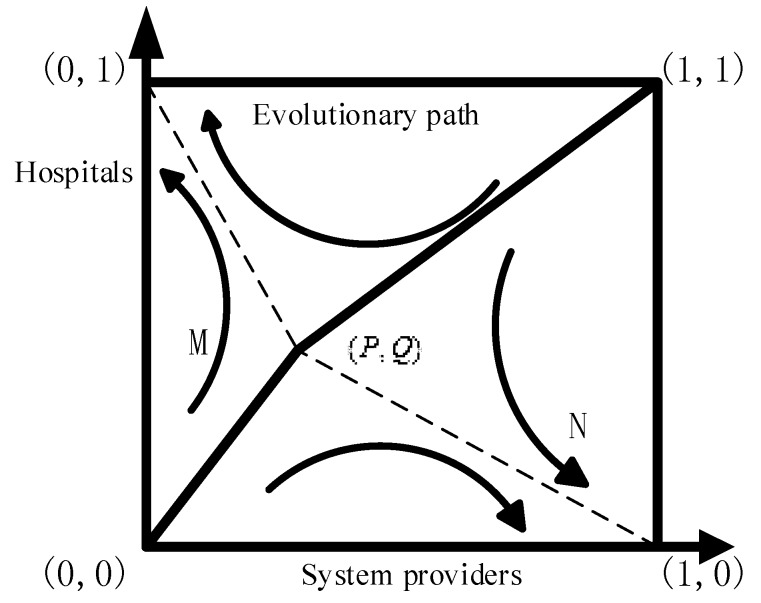
Evolutionary path of free riding.

In order to discuss the impacts of different parameters on ESS, we take Proposition 4 as an example. Based on the equilibrium analysis, the ESS of system providers and hospitals can be either (invest, not invest) or (not invest, invest) when free riding is present. As shown in Figure 9, the probability of choosing (invest, not invest) is greater if *S_M_* > *S_N_* while the probability of choosing (not invest, invest) is higher if *S_M_* < *S_N_*. *S_M_* can be defined as follows:

(27) $$S_{M}=\frac{1}{2}[\frac{\beta_{0}P_{H}-C_{H}}{\xi_{H}-(\beta_{1}-\beta_{0}+1)P_{H}}+\frac{\xi_{S}-(1+\alpha_{1})P_{S}+C_{S}}{\xi_{S}-(\alpha_{1}-\alpha_{0}+1)P_{S}}]$$

There are 10 variables (*P_S_*, *P_H_*, *ξ_S_*, *ξ_H_*, *C_S_*, *C_H_*, *α*_0_, *α*_1_, *β*_0_, *β*_1_) influencing the ESS. We take investment costs (*C_S_*, *C_H_*) as examples, and other scenarios are similar.

**Figure 10:**
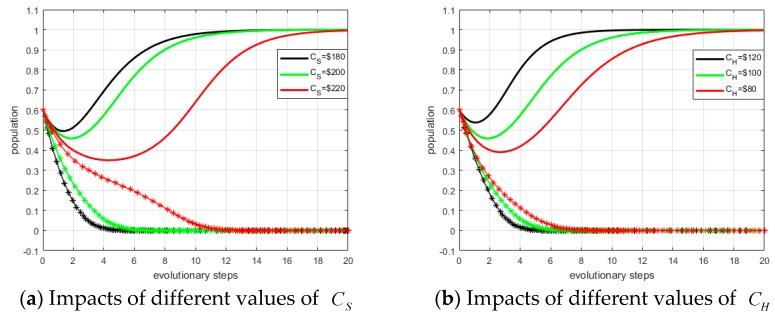
Impacts of investment costs on ESS.

First, we set the values of *C_S_* as: *C_S_* = $180, $200, and $220. Other values remain fixed as defined in Section 4.2. The evolutionary trends under differing values of variables can be compared and the simulation result is shown in panel (a) of Figure 10. The dotted lines represent system providers, and the solid lines represent hospitals. The ESS is (not invest, invest). From panel (a) in Figure 10, we observe that the smaller *C_S_*, the fewer steps to reach ESS.

Similarly, we define the values of *C_H_* as: *C_H_* = $80, $100, and $120. The simulation result is shown in in panel (b) of Figure 10. From panel (b) in Figure 10, we can conclude that the larger *C_H_* are, the higher probability of converging to (0, 1).

The impacts of other variables on ESS are shown in Table 9.

## 5. Implications and Countermeasures

To provide useful insights for the multiple participants in the privacy protection of mHealth system: (i) the system providers; (ii) the hospitals; (iii) the governments, we worked with Nanjing Drum Tower Hospital and Jiangsu Province Hospital of TCM, two famous hospitals with well-established mHealth systems in Nanjing, China. While building this evolutionary game theoretic model, we also interacted with Department of Health of Jiangsu Province, China, to help us understand the regulations involved in healthcare. Based on the model analysis and simulation results, we draw the conclusion that profit growth coefficients, investment costs, benefits from free riding and governmental regulation all play important roles in the investment choice of privacy protection. In particular, we find that if the profit growth coefficients are prohibitively small, system providers and hospitals will not invest in privacy protection, even if adequate government subsidies are available. Hence, we propose three different strategies for policy makers that can help boost participation in privacy investment.

(1) Increasing the minimum profit growth coefficients and reducing the investment costs

According to Proposition 1, system providers and hospitals will choose to “not invest” due to the relatively small benefits to be obtained from privacy investment, even if they may potentially receive a fine from governments. According to Proposition 9, to (invest, invest, not regulation) is the optimal state of privacy protection in a mHealth system. Ideally, system providers and hospitals will invest in privacy protection without the regulation of governments. Based on the above propositions, we find that the probability of privacy investment is positively related to the size of the profit growth coefficients. If the profit growth coefficients increase to a critical level, system providers and hospitals will obtain the expected benefits from investment in privacy protection, enabling patient privacy to be protected at a higher and more secure level. Therefore, increasing the minimum profit growth coefficients and reducing investment costs would help system providers and hospitals obtain larger benefits if and when they choose to “invest” in privacy protection. The policy makers can create these conditions by implementing the following measures:

- Support innovation of privacy protection technology. Any technological innovations related to privacy protection that can increase payoffs and reduce costs should be encouraged and motivated through National Science and Technology Plans or industrial development funds [62]. Governments should give priority to financially supporting or encouraging privacy protection R&D through policy incentives and financial subsidies.
- Enhance privacy awareness. Proper privacy education programs should be strengthened, in order to remove current forms of narrow-minded consciousness relating to privacy protection. Additionally, privacy protection lectures should be held, where experts in this domain would be invited to systematically explain to the citizens how to develop an appropriate attitude towards privacy protection.
- Provide two types of a mHealth service. Hospitals can provide two types of a mHealth service. The first type is a basic service, which patients could obtain it at a low price. The second is a value-added service, which would offer improved levels of patient safety and privacy. However, this service would be provided at a higher price. With improved of privacy awareness, patients would be willing to pay more for better privacy protection. Through this two-type method, system providers and hospitals could obtain the right balance between benefits and costs of privacy investment.

(2) Strengthening privacy advertisements and improving privacy regulations

According to Proposition 6, Proposition 7 and Proposition 8, when the profit growth coefficients and the investment costs of system providers and hospitals remain fixed, the probability of choosing to “invest” will be greater if governments choose to “regulate”. Based on the model analysis, the probability of governmental regulation is negatively related to regulation costs, and positively related to reputation profits. Positive public service advertisements unconsciously affect people’s behavior and thoughts, and shape their values. As such, advertising is an effective way to enhance reputation profits and reduce regulation costs. In addition, the absence of trust might be a severe issue and a major challenge to effective privacy protection in the healthcare domain. With serious doubts coming from the patients, a powerful legal system should be built, in order to improve patient trust levels and to reshape the credit mechanism rather than adopting industrial self-discipline. Therefore, we need to improve the relevant laws and regulations in China, in order to make clear the privacy authority of individuals and the responsibility of governments. In this way, governmental regulations can be properly implemented, and citizens can immediately preserve and defend their legal rights.

(3) Intensifying punishment and offering incentives

According to Proposition 1 to Proposition 5, if governments choose to “not regulate”, the cost of privacy investment is so high under current technical conditions that system providers and hospitals react negatively. Additionally, one important reason for the strategic choice to “not invest” (and free riding instead) is that the entities do not have to pay very much for their misdemeanors. Based on the model analysis, giving larger subsidies and fines to system providers and hospitals will increase the probability of privacy investment. On the one hand, governments should reward and support those entities that persist in implementing privacy protection. Governments should guide system providers and hospitals towards transforming their attitude toward investment to one that supports the enhancement of security and privacy awareness. On the other hand, because of the importance of rewarding and punishing mechanisms used under the current technical conditions, the power of multiple social organizations should be used to supplement the regulation of governments. This could include such steps as relaxing approval conditions, in order to give legality and authority to system providers and hospitals, and supporting a variety of privacy protection activities (organized by the associations) through financial subsidies and social donations.

## 6. Conclusions

Taking mHealth system as the context, we propose an evolutionary game-theoretic model to assess the decision making of privacy investment among system providers, hospitals and governments. We examine the conditions under which the chosen strategy is an evolutionary stable strategy, and investigate to quantify the appropriate investment of privacy investment and regulation. Then, we design a numerical simulation to explore and verify the theoretical results for managerial implication. Our model is generalizable to other similar healthcare systems and settings around the world, but not in those which are free or heavily state subsidized. We obtained the following results with potential implications via a theoretical analysis and simulation:

- The strategic choice of governmental regulation is mainly correlated with the size and degree of reputation profits and credit losses, as well as the cost of regulation. These factors profoundly affect the investment choice of system providers and hospitals.
- The strategic choices of privacy investment made by system providers and hospitals are not only correlated with the profits from investment, but those choices also affected by the extra benefits from free riding and the fine (or subsidy) from governments.
- If the profit growth coefficients are prohibitively small, then system providers and hospitals will choose to “not invest” due to the small benefits they will receive. This is true even if they receive a fine/subsidy from governments. When the profit growth coefficients increase to a critical level, the probability of choosing “invest” will be correspondingly larger if governments choose to “regulate”.
- As regulation costs increase, the strategic choice of governments will change from to “regulate” to “not regulate”. Similarly, as the profit growth coefficients increase, the strategy profile of system providers and hospitals will change as follows: (not invest, not invest), (not invest, invest), (invest, not invest), (invest, invest).
- If the extra benefits from free riding are large enough, the probability of system providers and hospitals choosing to “not invest” by will increase. On the other hand, if the fine/subsidy from governments increases, the probability of choosing to “not invest” and to free ride instead will decrease.
- If the profit growth coefficients are larger enough, system providers and hospitals will be willing to invest in privacy protection even if without governmental regulation. This result will not accrue additional benefits to system providers and hospitals, but will also reduce the cost to governments. Therefore, in reality the optimal stage of privacy protection is to (invest, invest, not regulate).

In this study, we use mHealth as the context for illustration, however, the development can be adapted with some effort to other emerging domains. Our study has several limitations that can be addressed in follow-up study. First, one could instead use an evolutionary game model for strategy choice based on a nonlinear demand function. It would be very interesting to compare those results with ours, but it would be very complicated to analyze due to its complexity. Second, a scenario involving an increased demand for patients’ privacy protection might be considered as this will affect the evolutionary path of the strategies. Third, not all the behavior of privacy investment or free riding can be evaluated precisely, for the sake of limited budgets and technological supports. How to enhance the accuracy of regulation on privacy investment should be addressed. Finally, an interesting issue to address in future work is how other factors (e.g., the advertising investment of system providers) affect the evolution of the choice of strategy for privacy protection.

## Figures and Tables

**Table 1:** Summary of game theoretic approaches to security & privacy problems.

Security & Privacy Problems	Game Model	Solution
Network attack-defense	Stochastic game	Nash equilibrium
Network security measurement	Static zero-sum game	Nash equilibrium
IDS configuration	Dynamic Bayesian game	Bayesian Nash equilibrium
Location privacy	Incomplete information static game	Bayesian Nash equilibrium
Security investment based on the relationship of attack-defense	Static game, Stackelberg game, dynamic Bayesian game	Nash equilibrium, Bayesian Nash equilibrium
Security investment based on the internal relationship of defenders	Differential game, repeated game, dynamic Bayesian game	Nash equilibrium, Bayesian Nash equilibrium, belief-based strategy
Security investment based on the relationship of multiple players	Incomplete information game, repeated game, dynamic Bayesian game	Nash equilibrium, Bayesian Nash equilibrium, belief-based strategy

**Table 2:** Key notations of evolutionary game model.

Notations	Connotations
*P_S_*	Profits of system providers if system providers and hospitals do not invest, *P_S_* > 0
*P_H_*	Profits of hospitals if system providers and hospitals do not invest, *P_H_* > 0
*R*_1_	Reputation profits of governments if system providers and hospitals choose to “invest”, *R*_1_ > 0
*R*_0_	Reputation profits of governments if they choose to “regulate”, and only one side of system providers and hospitals choose to “invest”, *R*_1_ > *R*_0_ > 0
*C_S_*	Investment costs of system providers, *C_S_* > 0
*C*	Investment costs of hospitals, *C_H_* > 0
*C_G_*	Regulation costs of governments, *C_G_* > 0
*L*	Credit loss incurred by governments if any of the system providers and hospitals choose to “not invest”, and governments choose to “not regulate”, *L* > 0
*F*	Fine for system providers and hospitals if they choose to “not invest”, *F* > 0
*ξ_S_*	Profits of system providers from free riding, *ξ_S_* > *P_S_* > 0
*ξ_H_*	Profits of hospitals from free riding, *ξ_H_* > *P_H_* > 0
*α*_0_	Profit growth coefficient of system providers if only system providers invest, *α*_0_ > 0
*α*_1_	Profit growth coefficient of system providers if both system providers and hospitals invest, *α*_1_ > *α*_0_ > 0
*β*_0_	Profit growth coefficient of hospitals if only hospitals invest, *β*_0_ > 0
*β*_1_	Profit growth coefficient of hospitals if both of system providers and hospitals invest, *β*_1_ > *β*_0_ > 0

**Table 3:** The payoff matrix.

Strategy			Payoffs		
Systems providers	Hospitals	Governments	Systems providers	Hospitals	Governments
invest	invest	regulate	$(1+\alpha_{1})P_{S}-C_{S}$	$(1+\beta_{1})P_{H}-C_{H}$	$R_{1}-C_{G}$
not invest	invest	regulate	$\xi_{S}-F$	$(1+\beta_{0})P_{H}-C_{H}+F$	$R_{0}-C_{G}$
invest	not invest	regulate	$(1+\alpha_{0})P_{S}-C_{S}+F$	$\xi_{H}-F$	$R_{0}-C_{G}$
not invest	not invest	regulate	$P_{S}-F$	$P_{H}-F$	$2F-C_{G}$
invest	invest	not regulate	$(1+\alpha_{1})P_{S}-C_{S}$	$(1+\beta_{1})P_{H}-C_{H}$	$R_{1}$
not invest	invest	not regulate	$\xi_{S}$	$(1+\beta_{0})P_{H}-C_{H}$	−L
invest	not invest	not regulate	$(1+\alpha_{0})P_{S}-C_{S}$	$\xi_{H}$	−L
not invest	not invest	not regulate	$P_{S}$	$P_{H}$	−L

**Table 4:** Values of equilibrium points.

Equilibrium Points	$a_{11}$	$a_{22}$	$a_{33}$
$P_{1}(0,0,0)$	$\alpha_{0}P_{S}-C_{S}$	$\beta_{0}P_{H}-C_{H}$	$2F+L-C_{G}$
$P_{2}(0,0,1)$	$\alpha_{0}P_{S}-C_{S}+2F$	$\beta_{0}P_{H}-C_{H}+2F$	$-(2F+L-C_{G})$
$P_{3}(0,1,0)$	$\alpha_{1}P_{S}-C_{S}-(\xi_{S}-P_{S})$	$-(\beta_{0}P_{H}-C_{H})$	$R_{0}+L-C_{G}$
$P_{4}(0,1,1)$	$\alpha_{1}P_{S}-C_{S}-(\xi_{S}-P_{S})+F$	$-(\beta_{0}P_{H}-C_{H}+2F)$	$-(R_{0}+L-C_{G})$
$P_{5}(1,0,0)$	$-(\alpha_{0}P_{S}-C_{S})$	$\beta_{1}P_{H}-C_{H}-(\xi_{H}-P_{H})$	$R_{0}+L-C_{G}$
$P_{6}(1,0,1)$	$-(\alpha_{0}P_{S}-C_{S}+2F)$	$\beta_{1}P_{H}-C_{H}-(\xi_{H}-P_{H})+F$	$-(R_{0}+L-C_{G})$
$P_{7}(1,1,0)$	$-[\alpha_{1}P_{S}-C_{S}-(\xi_{S}-P_{S})]$	$-[\beta_{1}P_{H}-C_{H}-(\xi_{H}-P_{H})]$	$-C_{G}$
$P_{8}(1,1,1)$	$-[\alpha_{1}P_{S}-C_{S}-(\xi_{S}-P_{S})+F]$	$-[\beta_{1}P_{H}-C_{H}-(\xi_{H}-P_{H})+F]$	$C_{G}$
$P_{9}(x*,y*,z*)$	0	0	0

**Table 5:** Different values of $C_{G}$, $R_{0}$, F, L and ESSs of governments.

$C_{G}$	$R_{0}$	L	F	ESS
$400	$200	$100	$40	not regulate
$250	$200	$100	$40	regulate if any one side of system providers and hospitals choose to “invest”
$80	$200	$100	$40	regulate

**Table 6:** Different values of *α*_0_, *α*_1_, *β*_0_, *β*_1_ and ESSs when *C_G_* > 2*R* + *L*.

$\alpha_{0}$	$\alpha_{1}$	$\beta_{0}$	$\beta_{1}$	ESS
0.2	0.4	0.1	0.3	(not invest, not invest, not regulate)
0.2	0.4	0.4	0.6	(not invest, invest, not regulate)
0.5	0.7	0.1	0.3	(invest, not invest, not regulate)
0.5	0.7	0.4	0.6	free riding
1.0	1.2	0.9	1.1	(invest, invest, not regulate)

**Table 7:** Different values of *α*_0_, *α*_1_, *β*_0_, *β*_1_ and ESSs when 2*F* + *L* < *C_G_* < *R*_0_ + *L*.

$\alpha_{0}$	$\alpha_{1}$	$\beta_{0}$	$\beta_{1}$	ESS
0.2	0.4	0.3	0.5	(not invest, invest, regulate)
0.4	0.6	0.1	0.3	(invest, not invest, regulate)
0.4	0.6	0.3	0.5	free riding

**Table 8:** Different values of *α*_0_, *α*_1_, *β*_0_ and *β*_1_ for sensitivity analysis.

	$\alpha_{0}$	$\alpha_{1}$	$\beta_{0}$	$\beta_{1}$
1	0.1	0.3	0.05	0.2
2	0.15	0.35	0.08	0.25
3	0.2	0.4	0.1	0.3
4	0.25	0.45	0.12	0.35
5	0.3	0.5	0.15	0.4

**Table 9:** Impacts on ESS when variables change.

Parameters Change	$S_{M}$ ($S_{N}$)	ESS
$\alpha_{0}$↓, $\alpha_{1}$↓	↑(↓)	(not invest, invest)
$\beta_{0}$↑, $\beta_{1}$↑	↑(↓)	(not invest, invest)
$P_{S}$↓	↑(↓)	(not invest, invest)
$P_{H}$↑	↑(↓)	(not invest, invest)
$C_{S}$↑	↑(↓)	(not invest, invest)
$C_{H}$↓	↑(↓)	(not invest, invest)
$\xi_{S}$↑	↑(↓)	(not invest, invest)
$\xi_{H}$↓	↑(↓)	(not invest, invest)
